# A Method for Measuring the Volume of Transdermally Extracted Interstitial Fluid by a Three-Electrode Skin Resistance Sensor

**DOI:** 10.3390/s140407084

**Published:** 2014-04-22

**Authors:** Dachao Li, Ridong Wang, Haixia Yu, Guoqing Li, Yue Sun, Wenshuai Liang, Kexin Xu

**Affiliations:** State Key Laboratory of Precision Measuring Technology and Instruments, Tianjin University, Tianjin 300072, China; E-Mails: rdwang@tju.edu.cn (R.W.); jyyhx2000@163.com (H.Y.); liguoqing@tju.edu.cn (G.L.); sy86311@gmail.com (Y.S.); liangwenshuai@tju.edu.cn (W.L.); kexin@tju.edu.cn (K.X.)

**Keywords:** blood glucose prediction, interstitial fluid, transdermal extraction, skin resistance sensor, volume measurement

## Abstract

It is difficult to accurately measure the volume of transdermally extracted interstitial fluid (ISF), which is important for improving blood glucose prediction accuracy. Skin resistance, which is a good indicator of skin permeability, can be used to determine the volume of extracted ISF. However, it is a challenge to realize *in vivo* longitudinal skin resistance measurements of microareas. In this study, a three-electrode sensor was presented for measuring single-point skin resistance *in vivo*, and a method for determining the volume of transdermally extracted ISF using this sensor was proposed. Skin resistance was measured under static and dynamic conditions. The correlation between the skin resistance and the permeation rate of transdermally extracted ISF was proven. The volume of transdermally extracted ISF was determined using skin resistance. Factors affecting the volume prediction accuracy of transdermally extracted ISF were discussed. This method is expected to improve the accuracy of blood glucose prediction, and is of great significance for the clinical application of minimally invasive blood glucose measurement.

## 1. Introduction

Minimally invasive blood glucose measurements based on the transdermal extraction and analysis of interstitial fluid (ISF) has gained the attention of researchers. This method determines the blood glucose level based on monitoring the glucose concentration in ISF because a close relation exists between the glucose concentration of the ISF and that of blood [1]. The advantages of this method include that this method is painless and that continuous monitoring can be realized [2]. There have been continuously increasing efforts in the last few decades in this field. The GlucoWatch G2 Biographer device (Cygnus Inc., Redwood City, CA, USA), which was the only system approved by the US FDA (Food and Drug Administration) for real-time readings, measures the ISF glucose concentration by a reverse iontophoresis-based extraction of ISF through the skin [3,4]. Due to the small volume of extracted ISF and the slow extraction speed, this method was limited by inaccurate glucose measurements if the patient was moving, exercising, sweating or experiencing rapid temperature changes. As a result, the device eventually failed [3]. Our research group proposed a method using ultrasound and vacuum to overcome the drawbacks of the GlucoWatch G2 Biographer device. Ultrasound was used to create micropores to increase the permittivity of the skin to ISF [5], and a vacuum pump was applied to enhance the convection of ISF. Then, ISF was transdermally extracted with higher fluxes using these two techniques. High fluxes of analytes were beneficial by having high detectable analyte concentrations and short collection times and by lowering interference from background analytes (*i.e.*, sweat) [6]. Although the transdermal ISF extraction technique using ultrasound and vacuum offered the promise of noninvasive, continuous, and real-time glucose monitoring, the extracted ISF, which scattered throughout the skin surface similar to dewdrops, was difficult to collect [7]. Thus, the ISF was diluted with a certain volume of buffer for easy collection in our research. Because the glucose concentration of diluted ISF can be obtained and because the volume of the buffer was known, the volume of transdermally extracted ISF was required for determining the glucose concentration of the ISF. However, the volumes of transdermally extracted ISF on the same position of a patient fluctuated with time using continuous glucose monitoring. For different positions of the same patient or for different patients, the volumes fluctuated more wildly. Although a microflow meter that was integrated into a microfluidic chip can be used to measure the volume of transdermally extracted ISF [8], liquid residue remained in the microchannels, which can affect the measurement results. Thus, until now, it has been difficult to realize *in situ* measurements of the accurate volume of transdermally extracted ISF.

Ultrasound is used to destroy the stratum corneum, and then vertical micropores are formed. Thus, the longitudinal skin resistance of the ultrasonic area is a good indicator of skin permeability [9]. However, it is a challenge to accurately measure the longitudinal skin resistance. Some possible approaches have been proposed to measure skin resistance. Martinsen *et al.* [10] proposed a system for live finger detection, which was based on simultaneous measurements of the electrical bio-impedance of different skin layers. Colbert *et al.* [11] proposed a single channel skin impedance system to measure the acupuncture point skin impedance. Both of these methods ignored the aeolotropy of skin, and the acquired skin resistance had no specific direction. Kinouchi *et al.* [12] proposed a fast *in vivo* method for the measurement of local tissue bio-impedance. The limitation of this method is that subcutaneously implanting a needle is invasive. Krishna *et al.* [13] proposed a multi-well resistance chamber to measure the electrical resistance of skin. This method could not realize *in vivo* measurements of skin resistance. In particular, these approaches, which ignored the interference of lateral skin resistance, cannot accurately obtain the longitudinal skin resistance.

In this paper, we present a three-electrode sensor to non-invasively realize *in vivo* longitudinal skin resistance measurements with high accuracy, which eliminates the interference of lateral skin resistance. Then, the volume of transdermally extracted ISF was measured using this sensor according to the relation between longitudinal skin resistance and skin permeability. The *in situ* measurement of the volume of transdermally extracted ISF was realized for the first time using this method.

## 2. Method of Measuring the Volume of Transdermally Extracted Interstitial Fluid (ISF)

### 2.1. Requirements for Measuring the Volume of Transdermally Extracted ISF for Blood Glucose Prediction

In the fasting state or 2 h after a meal, the relation of glucose between blood and ISF is linear [14]. Therefore, the blood glucose concentration (*CB_i_*) can be indirectly determined by extracting the ISF and monitoring the glucose concentration of ISF. To collect the minute volume of ISF on the skin surface after transdermal extraction, a defined volume of phosphate buffer was used to dilute the transdermally extracted ISF. Then, the volume of diluted ISF was large enough to be easily collected. Based on the conservation of the amount of glucose before and after dilution, the following equation can be obtained:

(1) $$CB_{i}\cdot x_{i}=k\cdot CI_{i}\cdot x_{i}=k\cdot (V+x_{i})\cdot CS_{i}$$

(2) $$x_{i}=P_{i}\cdot t\cdot A$$

where *CS_i_* was the glucose concentration of the diluted sample solution, which can be measured using a glucose sensor; *V* was the volume of phosphate buffer injected into the extraction chamber (50 μL); *x_i_* was the volume of transdermally extracted ISF; *k* was a coefficient that reflected the relation between the blood glucose concentration and the ISF glucose concentration of a specific person under normal physiological conditions; *P_i_* was the permeation rate of ISF in the skin; *t* was the extraction time (10 min); and *A* was the skin area used for the ISF extraction (0.785 cm^2^). The following equation:

(3) $$CB_{i}=k\cdot \left(1+\frac{V}{x_{i}}\right)\cdot CS_{i}=k\cdot \left(1+\frac{V}{P_{i}\cdot t\cdot A}\right)\cdot CS_{i}$$

was used to predict the blood glucose concentration based on the glucose concentration of the diluted ISF. As previously mentioned, *V*, *t*, and *A* were already known; *CS_i_* and *k* were measurable. Thus, the key to predicting the blood glucose concentration was monitoring the volume of transdermally extracted ISF, *x_i_*, or the permeation rate of ISF, *P_i_*. Because the value of *x_i_* or *P_i_* was too small to be directly monitored, a method that used a parameter that can be easily measured and that had a simple relation with *x_i_* or *P_i_* was required to indirectly determine the blood glucose concentration.

### 2.2. Using Skin Resistance to Measure the Volume of Transdermally Extracted ISF

Compared with the volume of transdermally extracted ISF, the resistance or conductance of the skin along the penetration direction can be monitored more easily. Additionally, Mitragotri *et al.* showed that skin conductance is an excellent indicator of skin permeability [9]. For hydrophilic solutes, the transdermal transmission path is similar to that for charged ions, and the permeability characteristics are closely associated with skin conductivity. The following relation between *P_i_* (the permeation rate of ISF in skin) and skin conductivity was demonstrated by Tang *et al.* [15]:

(4) $$P_{i}=C\frac{\sigma }{\Delta x}$$

where *C* was a constant related to the size of skin pores, the inherent characteristics of the electrolyte solution, the permeating substance, and the charged ions; σ was the skin conductivity; and Δ*x* was the thickness of the stratum corneum.

Considering the following relation:

(5) $$\frac{\sigma }{\Delta x}=\frac{G_{i}}{A}=\frac{1}{A\cdot R_{i}}$$

where *G* and *R* were the conductance and resistance of the skin with an area *A* and thickness *Δx*, respectively, from Equations (4) and (5), we obtained the following relations:

(6) $$P_{i}=C\frac{G_{i}}{A}$$

or:

(7) $$P_{i}=\frac{C}{A\cdot R_{i}}$$

Thus, from Equations (2), (6), and (7), we obtained the following equation:

(8) $$x_{i}=C\cdot \frac{G_{i}}{A}\cdot t\cdot A=C\cdot t\cdot G_{i}=C_{1}G_{i}$$

or:

(9) $$x_{i}=\frac{C}{A\cdot R_{i}}\cdot t\cdot A=\frac{C\cdot t}{R_{i}}=\frac{C_{1}}{R_{i}}$$

Thus, a quantitative relationship between the volume of transdermally extracted ISF and the conductance or resistance of skin was established. By substituting this relation into Equation (3), the blood glucose prediction is achieved as follows:

(10) $$CB_{i}=k\cdot \left(1+\frac{V}{C_{1}G_{i}}\right)=k\cdot \left(1+\frac{V}{C_{1}}R_{i}\right)\cdot CS_{i}$$

To determine *C*_1_, the blood glucose concentration was measured using a portable blood glucose meter when ISF was transdermally extracted for the first time. The glucose concentration of the diluted ISF and the skin resistance of the extracting area were also acquired. *C*_1_ was obtained by substituting these values into Equation (10), together with the known value *V*. When *C*_1_ was determined, this value can remain stable for a long time if the outside and skin conditions were controlled properly. During the subsequent extraction process, the volume of transdermally extracted ISF can be easily determined based on Equations (8) and (9) if the skin resistance or skin conductance was obtained. Combining the glucose concentration of the diluted ISF, the blood glucose concentration can be accurately predicted using Equation (10). Thus, Equation (10), which was used to predict the blood glucose concentration by considering the volume of extracted ISF, was the key equation. The volume of extracted ISF was reflected using the longitudinal skin resistance in the equation.

## 3. Design of the Three-Electrode Sensor for Skin Resistance Measurement

The *in vitro* resistance of skin can be directly measured using electrodes applied to both sides of the skin. Obviously, this method was not appropriate for measuring *in vivo* skin resistance because the electrodes must be invasively implanted [12], which was contrary to the original intention of minimally invasive blood glucose monitoring. A three-electrode sensor was designed to measure the skin resistance at the ISF extraction point.

**Figure 1.**
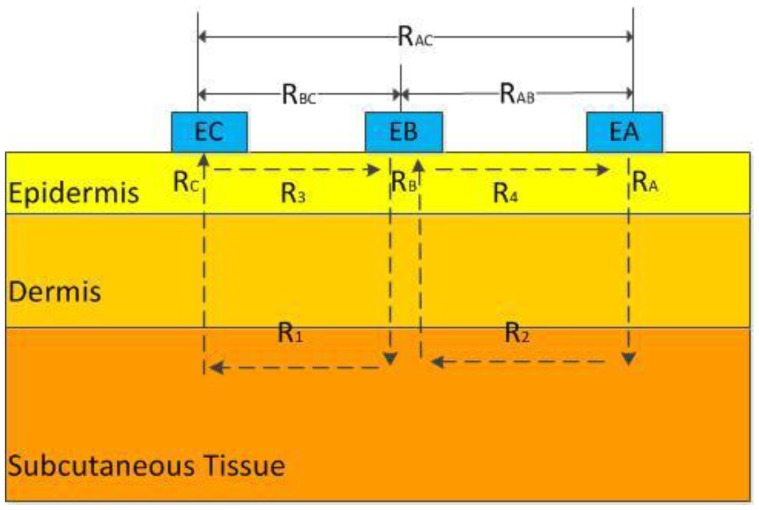
Three-electrode sensor.

To simplify the analysis, the skin was treated as a pure resistance model. The structure of the three-electrode sensor was shown in Figure 1 (*E*_A_, *E*_B_, and *E*_C_ are electrodes A, B, and C, respectively) [16]. The area of skin to be measured was under electrode B. *R*_A_, *R*_B_, and *R*_C_ were the longitudinal resistances of the epidermis under electrodes A, B, and C, respectively; *R*_1_ and *R*_2_ were the resistances of the tissue under the epidermis between electrodes B and C and between electrodes A and B, respectively. *R*_3_ and *R*_4_ were the resistances of the epidermis between electrodes B and C and between electrodes A and B, respectively. When a voltage was applied between two electrodes, the main path of the current originated from one electrode, penetrated the epidermis, was transmitted to the deep tissue, and reached the other electrode by penetrating the epidermis again. Then, the following relations were established:

(11) $$R_{\text{AB}}=\frac{\left(R_{\text{A}}+R_{2}+R_{\text{B}}\right)\cdot R_{4}}{\left(R_{\text{A}}+R_{2}+R_{\text{B}}\right)+R_{4}}$$

(12) $$R_{\text{BC}}=\frac{\left(R_{\text{B}}+R_{1}+R_{\text{C}}\right)\cdot R_{3}}{\left(R_{\text{B}}+R_{1}+R_{\text{C}}\right)+R_{3}}$$

(13) $$R_{\text{AC}}=\frac{\left(R_{\text{A}}+R_{2}+R_{1}+R_{\text{C}}\right)\cdot \left(R_{3}+R_{4}\right)}{\left(R_{\text{A}}+R_{2}+R_{1}+R_{\text{C}}\right)+\left(R_{3}+R_{4}\right)}$$

*R*_3_ and *R*_4_ were extremely large, so the following relations were established:

(14) $$R_{AB}=R_{A}+R_{2}+R_{B}$$

(15) $$R_{BC}=R_{B}+R_{1}+R_{C}$$

(16) $$R_{AC}=R_{A}+R_{2}+R_{1}+R_{C}$$

(17) $$R_{\text{B}}=\frac{R_{\text{AB}}+R_{\text{BC}}-R_{\text{AC}}}{2}$$

According to Equation (17), the longitudinal resistance of the epidermis under electrode B, which corresponded to the physical skin resistance along the permeating direction, can be calculated after *R*_AB_, *R*_BC_, and *R*_AC_ were measured. Then, the obtained longitudinal skin resistance, which eliminated the interference of lateral skin resistance, was used in Equation (10) to predict the blood glucose concentration.

## 4. Experiments

### 4.1. ISF Extraction and Collection

Ultrasound at 55 kHz was applied for approximately 30 s. The ultrasonic skin permeation device delivered ultrasonic energy to the skin through an aqueous ultrasound coupling medium. The application of low-frequency ultrasound (20–100 kHz) appeared to induce cavitation inside and outside the skin. Cavitation occurring at either location may cause the disordering of the *stratum corneum* (SC) lipids. In addition, oscillations of cavitation bubbles may result in significant water penetration into the disordered lipid regions. This situation may cause the formation of aqueous channels through the intercellular lipids of the SC. This formation allowed permeants to be transported across the disordered lipid domains, and then across keratinocytes and across the entire SC.

After skin permeation, a flanged glass cylinder (diameter 10 mm, height 20 mm) was fixed on the ultrasonically permeated target site. The chamber was filled with 50 μL of phosphate-buffered saline (PBS). The extraction was performed by a 10-min application of vacuum (10 in Hg) using an oil-less vacuum-pressure pump. At the end of extraction, the chamber contents were collected to measure the glucose concentration using an immobilized enzyme biological sensing analyzer (SBA-40C, Key Laboratory of Biosensor in Shandong Province, Jinan, China). The analyzer was a commercial device for glucose concentration measurement.

### 4.2. Skin Resistance Measuring System

**Figure 2.**
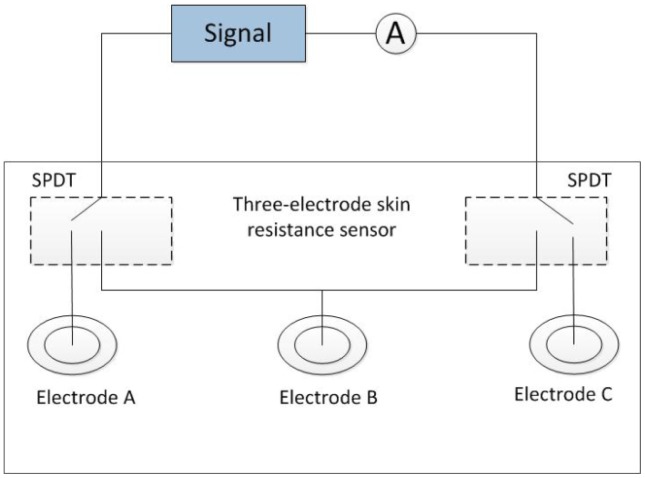
Skin resistance measuring system.

The skin resistance measuring system was shown in Figure 2. An alternating voltage (100 mV, 10 Hz) was generated from a signal source. Two single-pole double-throw (SPDT) switches were applied for two of the three electrodes. The weak current flowing through the skin between each of the two electrodes was measured using a picoammeter. The resistance between the two electrodes was obtained using Ohm's law. Disposable electrocardiography (ECG) electrodes were used for electrodes A and C. A disposable ECG electrode or an Ag-AgCl disc electrode was used for electrode B.

For measuring the skin resistance, an arbitrary function generator (AFG3022; Tektronix, Beaverton, OR, USA) was used as the signal source, and a picoammeter (6485; Keithley, Cleveland, OH, USA) was used to measure the weak current. The three electrodes (A, B and C) were arranged on the inside of the forearm. Electrode B was located on the ISF extraction point. Electrodes A and C were set on the wrist side and elbow side, respectively, of electrode B at the same distance away. The skin resistance of the ISF permeating area was obtained by calculating (*R_AB_* + *R_BC_* − *R_AC_*)/2, where *R_AB_*, *R_BC_*, and *R_AC_* were the resistances between each of the two electrodes, respectively. The skin area under electrode B was pretreated by low-frequency ultrasound for skin permeabilization. The extraction areas under electrode A and C were treated similarly to ensure that their resistances were at the same level, thereby improving the reliability of the results.

### 4.3. Skin Resistance Measuring Process

**Figure 3.**
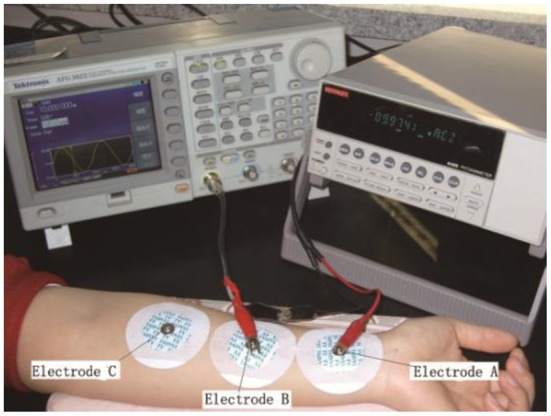
Skin resistance measurement based on a three-electrode system (static measurement).

To optimize this method, skin resistance was measured in two modes: static and dynamic. The static skin resistance measurement referred to the measurement of skin resistance before and after an extraction process. Due to their good stability and convenience, disposable ECG electrodes, which were made of Ag/AgCl, were used for electrodes A, B, and C (Figure 3). The average of the measurement results before and after extraction was considered the static skin resistance of this extraction process.

**Figure 4.**
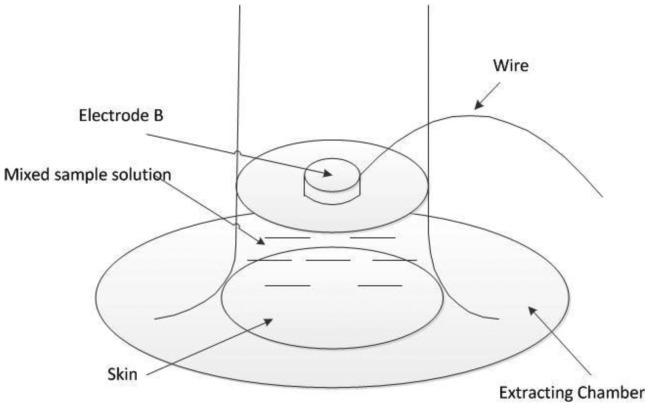
Electrode used for the dynamic measurement of skin resistance.

Dynamic skin resistance measurement referred to the measurement of skin resistance during the extraction process. Electrodes A and C were set as the static mode. Because the extraction chamber was small, the ECG electrode cannot fit into the chamber. An Ag/AgCl disc electrode was chosen as electrode B, which was placed in the extraction chamber facing down and immersed in the diluted ISF. Then, the current can be transmitted through electrode B and the sample solution into the skin. The wire connected to electrode B was led out of the chamber through a sealed hole on the wall of the chamber (Figure 4). To obtain the longitudinal skin resistance under electrode B, the resistances between A and B, between B and C, and between A and C were measured. During the extraction process, skin resistance was measured for five times with the same time interval (2 min). The average of the five values was considered the dynamic skin resistance of this extraction process.

## 5. Results and Discussion

### 5.1. Measuring Results of Skin Resistance

**Figure 5.**
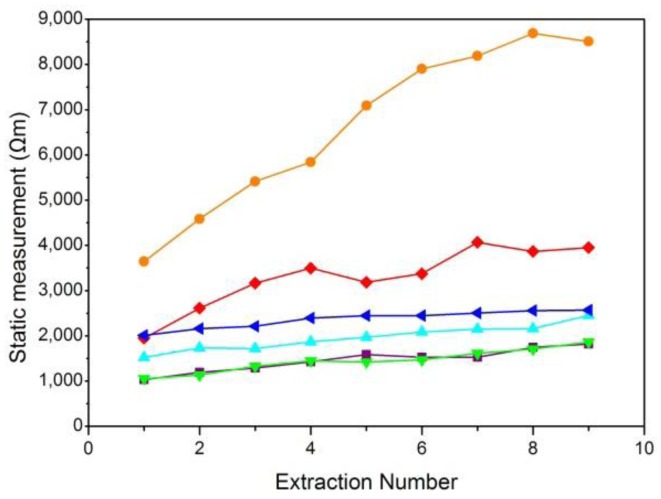
Results of six static skin resistance measurements. The time interval between each two measurement results was over 10 min.

**Figure 6.**
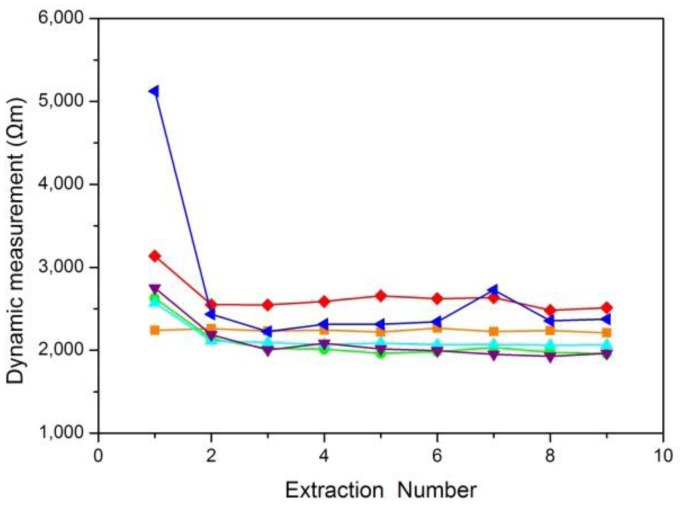
Results of six dynamic skin resistance measurements. The time interval between each two measurement results was over 10 min.

A skin resistance curve was drawn according to the average values of the skin resistance obtained from several ISF extractions. Six typical curves for static skin resistance measurements and six typical curves for dynamic skin resistance measurements were shown in Figures 5 and 6, respectively.

Figure 5 shows that the results of static skin resistance measurements between groups were quite different. Due to the recovery of the skin, the static resistance values in the same group were varied. However, the orange curve of Figure 5 had a relatively large range of variation, which should be considered the gross error. Compared with static skin resistance measurements, the results of dynamic skin resistance measurements (Figure 6) had little difference between groups. The dynamic resistance values in the same group reached a stable level within a short time. This process was generally accomplished during the first extraction. The cause of the great differences between the two measuring methods included the change in the skin state during the measuring process, the distinction between electrode B for the two modes, and the proportion of epidermal resistance in the acquired values.

The skin resistance we want to measure was epidermal resistance, which plays a key role in the barrier properties of skin. For dynamic skin resistance measurements, electrode B contacted the skin through the electrolyte solution. During the ISF extraction process, the electrolyte solution infiltrated into the epidermis, causing the resistance to greatly decrease within a short time. Thus, dynamic measurements cannot truly reflect the variation in skin penetration ability. Although the static skin resistance measurement was not as good as the dynamic skin resistance measurement for real-time testing, the epidermal resistance measured in the static mode was relatively larger and occupies the majority of the resistance values. Therefore, the static measurement was more consistent with our resistance measuring requirements.

### 5.2. Validation of this Method for Measuring the Volume of Transdermally Extracted ISF

The scientific authenticity of the derivation method for the volume of transdermally extracted ISF was examined using the results of skin resistance measurements. The linear relation between *P_i_* and *G_i_*/*A* in Equation (7) was the basis for this method. For the convenience of experimental observation, the following relation was considered. When the dilution factor of the ISF was large enough, *i.e.*, *V* ≫ *x_i_*, then Equation (3) can be simplified as follows:

(18) $$CB_{i}=\frac{V}{P_{i}\cdot t\cdot A}CS_{i}$$

(19) $$P_{i}=\frac{V}{CB_{i}\cdot t\cdot A}CS_{i}$$

When the blood glucose concentration *CB_i_* remained relatively stable, then *P_i_* ∝ *CS_i_*. The validation of the linear relation between *P_i_* and *G_i_*/*A* in Equation (6) converted to the linear relation between *CS_i_* and *G_i_*/*A*, where the extraction area *A* was given, and the values of *CS_i_* and *G_i_* can be directly measured.

**Figure 7.**
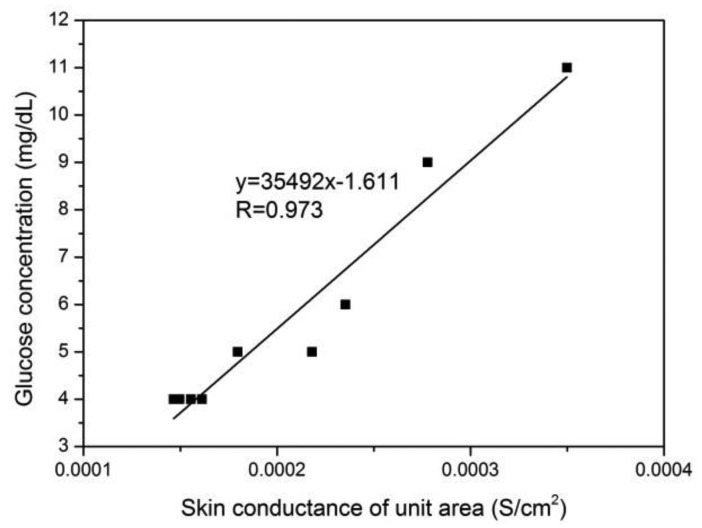
Relation between the glucose concentration of the sample solution and skin conductance.

The relation between skin conductance in a unit area (*G_i_*/*A*) and the glucose concentration of a sample solution (*CS_i_*) is shown in Figure 7. The glucose concentration of the sample solution was measured by an immobilized enzyme biological sensing analyzer, as previously mentioned, and the skin conductance in a unit area was calculated using the static skin resistance measurement value.

The linear relation between *P_i_* and *G_i_*/*A* in Equation (7) was proven by the high correlation coefficient between *CS_i_* and *G_i_*/*A*, as shown in Figure 7. Thus, the scientific authenticity of the derivation method for the volume of transdermally extracted ISF was validated.

### 5.3. Factors Affecting the Volume Prediction Accuracy of Transdermally Extracted ISF

According to Equation (10), factors affecting the volume prediction accuracy of transdermally extracted ISF included the factors that affect the measurement of skin resistance *R_i_* and the determination of the constant *C*_1_. The contact resistance [17] and the state (such as humidity and temperature) of the skin affected the results of static skin resistance measurement. Because *C*_1_ was closely related to the outside and skin conditions, it was crucial to stably maintain these conditions. To determine *C*_1_, the blood glucose concentration when ISF was transdermally extracted for the first time and the glucose concentration of the diluted ISF were required. The blood glucose concentration was acquired using a portable blood glucose meter, and the diluted ISF glucose concentration was acquired using a glucose biosensor. The measuring accuracies of the glucose meter and biosensor had great influence on the volume prediction accuracy.

To improve the volume prediction accuracy, the contact resistance should be considered, and the state of the skin should be properly controlled. This experiment can be conducted in a constant temperature environment, and a glucose meter with high precision can be used to monitor the blood glucose concentration. A more advanced glucose biosensor can also be developed to more accurately measure the diluted ISF glucose concentration.

## 6. Conclusions

A three-electrode sensor to measure the longitudinal skin resistance *in vivo* was presented. A method for determining the volume of transdermally extracted ISF using this sensor was proposed. Skin resistance was assessed using static and dynamic measurements. The characteristics of these two measurement modes were evaluated. The linear relation between skin conductance and the rate at which ISF permeated skin was verified. The feasibility of using skin resistance to deduce the volume of transdermally extracted ISF was proven. Finally, factors affecting the volume prediction accuracy of transdermally extracted ISF were analyzed and some possible improvement methods were given. This method provides a feasible way to accurately predict blood glucose concentrations in continuous glucose monitoring. For this paper, we primarily focused on the feasibility and stability of the method, which measured the longitudinal skin resistance using a three-electrode sensor and then determines the volume of transdermally extracted ISF using the longitudinal skin resistance. Future efforts will be concentrated on applying this method for measuring the glucose concentration of transdermally extracted ISF to improve the prediction accuracy of blood glucose concentration measurements.
